# Artificial Intelligence in the Discovery of Deep Eutectic Solvents with Lubricant Applications

**DOI:** 10.1021/acsomega.5c05944

**Published:** 2025-09-10

**Authors:** João P. Santos, Filipe H. B. Sosa, Dinis O. Abranches, João A. P. Coutinho

**Affiliations:** CICECO−Aveiro Institute of Materials, Department of Chemistry, 426216 University of Aveiro, Aveiro 3810-193, Portugal

## Abstract

Following evidence suggesting that deep eutectic solvents (DESs) can potentially replace conventional mineral-based lubricants, this study aims to leverage artificial intelligence to discover, and then experimentally prepare and characterize, novel DES-based lubricants. To do so, Gaussian processes (GPs) were employed to describe and predict relevant physicochemical properties of DESs, specifically density, viscosity, and melting temperature. This was accomplished by using a comprehensive data set encompassing nearly 400 different binary and ternary DESs and including 3985, 4197, and 2003 independent data points (different DES compositions and temperatures) for density, viscosity, and melting temperature, respectively. GPs were trained and rigorously evaluated, attaining testing set coefficients of determination of 0.98, 0.92, and 0.94, respectively. GPs were then used to predict the density, viscosity, and melting temperature of all possible binary 1:1 combinations of DES precursors available in the database, yielding more than 50,000 DESs. These DESs with precursors available in our laboratory and that were predicted to be liquid at room temperature, exhibiting either minimal density and minimal viscosity, or maximal density and maximal viscosity, were experimentally prepared and characterized. Good agreement was found between GP predictions and experimental results. Given the identification of DESs with exceptionally low viscosities, a subset of these liquids was selected for tribological evaluation. Finally, tribological tests revealed that several of the tested DESs, such as camphor:octanoic acid, outperformed the reference oil in terms of friction reduction.

## Graphical abstract

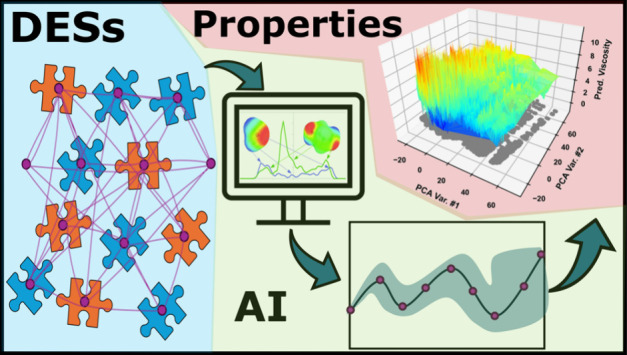

## 1 Introduction

Deep eutectic solvents (DESs) are liquid mixtures prepared by physically mixing solid compounds, typically one hydrogen bond donor (HBD) and one hydrogen bond acceptor (HBA).^1^ These solvents are characterized by strong cross-component hydrogen bonding and have garnered significant attention in recent years as innovative and environmentally friendly alternatives to traditional organic solvents.^2,3^ Their appeal lies both in their sustainable character and tunability, which can be achieved by adjusting the nature and relative compositions of their precursors. DESs have found a wide range of applications, including, among many other examples, metal extraction and separation,^4^ nanotechnology,^5^ carbon capture,^6^ polymer synthesis,^7^ and drug formulation.^8^

Lubricants are fluids used to reduce friction between moving surfaces, thus minimizing wear and improving the efficiency, reliability, and longevity of machinery and equipment. They form a protective layer between solid components, preventing direct contact and also aiding in heat dissipation. Surprisingly and despite being typically studied and employed as solvents, DESs have only recently found applications as lubricants.^9–15^ Given their potential for low toxicity, high biodegradability, and overall green nature, together with their high tunability, DESs present a promising alternative to conventional mineral-based lubricants. Their potential to replace traditional lubricants could lead to more environmentally friendly and efficient lubrication solutions in various engineering applications, joining the growing list of other green and sustainable candidates.^16^

Regardless of application, designing DESs is a complex task due to the vast array of available precursor candidates and relative compositions to choose from. Moreover, the physicochemical properties of DESs are not merely the weighted average of those of their precursors. This is well-known for their melting temperatures, which exhibit the typical V-shape of eutectic-type solid–liquid equilibrium phase diagrams, but extends to various other properties. For example, the biodegradability and toxicity of a DES cannot be directly inferred from its precursors due to potential synergistic and antagonistic effects.^17^ Additionally, DES properties are often modulated by incorporating additives. For instance, viscosity is typically lowered by adding a third, low molecular weight compound, such as water.^18^ As such, alternative design approaches beyond simple trial-and-error methods are needed to fully exploit the advantages of DESs.

Machine learning (ML) encompasses a collection of techniques that enable the efficient and accurate correlation of input (features) and output (labels) variables.^19^ Due to their unique ability to relate information and emulate complex feature-label relationships, ML models are rapidly becoming crucial in the development of novel materials, including the design of DESs.^20–23^ Despite their usefulness, training, tuning, and deploying ML models, particularly neural networks, often requires vast amounts of experimental data and dedicated computational resources. Gaussian Processes (GPs), a class of stochastic and nonparametric ML models, have recently demonstrated superior predictive accuracy and generalizability compared to neural networks in predicting the physicochemical properties of materials.^24^ Unlike neural networks, GPs can be fitted and applied without specialized computational resources, with hyperparameter tuning and training times that can reach under 1 min on a regular personal computer.

The GP models referenced in the previous paragraph rely on sigma profiles as features to describe molecules, thus converting molecular structures into numerical vectors that can be processed and understood by machine learning algorithms. Within the framework of the thermodynamic model COSMO-RS,^25^ sigma surfaces represent the polarity surfaces of molecules, tessellated into small surface area patches, each assigned a charge density (sigma). Sigma profiles are the unnormalized histograms of these sigma surfaces. By capturing and encoding surface charge distributions, sigma profiles effectively encapsulate the polarity of molecules and their potential for intermolecular interactions, namely through hydrogen bonding and van der Waals forces. Thus, sigma profiles offer a concise yet comprehensive representation of molecular behavior in various environments, making them an ideal feature to describe highly hydrogen-bonded fluids and mixtures such as DESs.

Inspired by the recent success of machine learning in describing the physicochemical properties of pure materials using sigma profiles and GP models, along with mounting evidence that DESs may serve as suitable alternatives to traditional mineral-based lubricants, this work has two primary objectives. First, GP models were developed to predict various physicochemical properties of DESs relevant to lubricant applications, specifically density, viscosity, and melting temperature. To do so, an extensive database containing both binary and ternary DES combinations at different compositions was taken from the literature. Then, these models were used to discover novel DES lubricants, with the density, viscosity, and friction coefficient of the best available candidates being experimentally assessed.

## 2 Materials and Methods

This section details the computational and experimental methods used in this work, including the computation of sigma profiles, the training of GP models, and the experimental characterization of novel DESs. The data sets and Python code employed are available in the following GitHub repository: https://github.com/dinisAbranches/AI_DES_Lubricants.

### 2.1 Sigma Profiles

As explained in the Section 1, sigma profiles encode the polarity surface of molecules in a two-dimensional fashion. More precisely, they are defined as^25^

$$P\left(\sigma \right)\cdot A=\frac{A_{i}\left(\sigma \right)}{A}\cdot A$$ 1

where *A* is the total surface area of a molecule, *A*_*i*_(σ) is the area of the surface segment *i* with screened charge σ, and *P*(σ) is the probability of finding a surface segment with screened charge σ. Note that the term *sigma profile* can be used to denote either *P*(σ) alone or the full *P*(σ) · *A* term (left-hand side of eq 1), with the latter definition being adopted in this work.

The software package TURBOMOLE^26^ was employed in this work to perform all necessary quantum chemistry calculations to obtain sigma profiles. To do so, each molecule of interest was embedded in the COSMO continuum solvation model with infinite permittivity and optimized using DFT with the BP-86 functional and the def-TZVP basis set. During each geometry cycle, the van der Waals surface of the molecule was constructed, tessellated, and screening charges computed for each area segment. This is the standard COSMO-RS procedure to compute sigma surfaces and was adopted in this work. Then, the software COSMOtherm^27^ was used to convert the sigma surfaces to sigma profiles, which involves an averaging and binning procedure to build the final unnormalized histograms, i.e., sigma profiles.^28–30^

Using the aforementioned procedure, the sigma profiles of all 342 individual DES precursors examined in this work were computed. These are depicted in Figure S1. Sigma profiles for DESs were then obtained by concatenating the sigma profiles of each individual precursor, weighted by its corresponding mole fraction:

$$\mathrm{SP}=[{\mathrm{SP}}_{1}x_{1},{\mathrm{SP}}_{2}x_{2},{\mathrm{SP}}_{3}x_{3}]$$ 2

where SP represents the concatenated sigma profile of a DES (a vector of size 183), SP_*i*_ represents the sigma profile of an individual DES component (vector of size 61), and *x*_*i*_ is its mole fraction.

### 2.2 Gaussian Processes

Within the framework of GPs, labels (*y*_*i*_) are assumed to be random variables that follow a joint Gaussian probability distribution:^31^

$$\left[\begin{array}{c} y_{1}\\ \vdots \\ y_{N} \end{array}\right]∼N\left(\left[\begin{array}{c} \mu_{1}\left(x_{1}\right)\\ \vdots \\ \mu_{N}\left(x_{N}\right) \end{array}\right],\left[\begin{array}{ccc} k_{11}\left(x_{1},x_{1}\right) & \cdots & k_{1N}\left(x_{1},x_{N}\right)\\ \vdots & \ddots & \vdots \\ k_{N1}\left(x_{N},x_{1}\right) & \cdots & k_{NN}\left(x_{N},x_{N}\right) \end{array}\right]\right)$$ 3

where *x*_*i*_ represents the feature (input) associated with label (output) *y*_*i*_, μ_*i*_ represents some mean function, and *k*_*ij*_ represents some covariance (kernel) function. In this work, *x*_*i*_ is the sigma profile of DES *i* (a vector containing 183 entries, as defined in Section 2.1) concatenated with the temperature of the system (only in the cases of density and viscosity, yielding a vector with 184 entries), while *y*_*i*_ is a physicochemical property (density, viscosity, or melting temperature).

Given a training data set containing *N* data points of the type (*x*_*i*_, *y*_*i*_), predictions for a new testing point (x_*_, y_*_) can be made by considering the following joint distribution:

$$\left[\begin{array}{c} y_{1}\\ \vdots \\ y_{N}\\ y_{*} \end{array}\right]∼N\left(\begin{array}{c} \mu =\left[\begin{array}{c} \mu_{1}\left(x_{1}\right)\\ \vdots \\ \mu_{N}\left(x_{N}\right) \end{array}\right]\\ \mu_{*}\left(x_{*}\right) \end{array},\left[\begin{array}{cc} \Sigma =\left[\begin{array}{ccc} k_{11}\left(x_{1},x_{1}\right) & \cdots & k_{1N}\left(x_{1},x_{N}\right)\\ \vdots & \ddots & \vdots \\ k_{N1}\left(x_{N},x_{1}\right) & \cdots & k_{NN}\left(x_{N},x_{N}\right) \end{array}\right] & \Sigma_{*}=\left[\begin{array}{c} k_{1*}\left(x_{1},x_{*}\right)\\ \vdots \\ k_{N*}\left(x_{N},x_{*}\right) \end{array}\right]\\ \Sigma_{*}^{T}=\left[k_{*1}\left(x_{*},x_{1}\right)\cdots k_{*N}\left(x_{*},x_{N}\right)\right] & \Sigma_{**}=k_{**}\left(x_{*},x_{*}\right) \end{array}\right]\right)$$ 4

Note how the covariance matrix is partitioned into four distinct blocks, namely **Σ** containing covariance among training data, Σ_*_ and Σ_*_^T^ containing the covariance between training and testing data, and Σ_**_ containing the covariance of the testing point. Likewise, the mean vector is sectioned into **μ** and μ_*_. Considering eq 4, predicting y_*_ becomes a matter of conditional probability, such that

$$y_{*}|Y∼N\left(\mu^{\prime },\Sigma^{\prime }\right)$$ 5

with $Y={\left[\begin{array}{ccc} y_{1} & \cdots & y_{N} \end{array}\right]}^{T}$ and:

$$\mu^{\prime }=\mu_{*}+\Sigma_{*}^{T}\Sigma^{-1}\left(Y-\mu \right)$$ 6

$$\Sigma^{\prime }=\Sigma_{**}-\Sigma_{*}^{T}\Sigma^{-1}\Sigma_{*}$$ 7

In this context, μ′ represents the GP-predicted value for y_*_, and Σ′ represents its corresponding, GP-predicted uncertainty.

To facilitate the implementation of GPs, employing a null mean function (μ­(*x*_*i*_) = 0) is a common choice in the literature, which was adopted in this work. Several popular kernels were tested in this work, namely the radial basis function kernel (RBF), the rational quadratic kernel (RQ), and the Matérn 3/2 kernel (M32):^32,33^

$$k_{\mathrm{RBF}}(x_{i},x_{j})=\sigma^{2}\exp\left(-\frac{∥x_{i}-x_{j}{∥}^{2}}{2l^{2}}\right)$$ 8

$$k_{\mathrm{RQ}}(x_{i},x_{j})=\sigma^{2}{\left(1+\frac{∥x_{i}-x_{j}{∥}^{2}}{2\alpha l^{2}}\right)}^{-\alpha }$$ 9

$$k_{M32}(x_{i},x_{j})=\sigma^{2}\left(1+\sqrt{3}\frac{∥x_{i}-x_{j}∥}{l}\right)\exp\left(-\sqrt{3}\frac{∥x_{i}-x_{j}∥}{l}\right)$$ 10

where ∥*x*_*i*_–*x*_*j*_∥ represents the Euclidean distance between points *x*_*i*_ and *x*_*j*_, σ^2^and *l* are the variance and length scale hyperparameters of the kernel, and α is a RQ-specific hyperparameter.

GPs can account for experimental uncertainty by supplementing the kernel of choice (see paragraph above) with a White noise kernel, defined as

$$k_{W}(x_{i},x_{j})=\delta (i,j)\sigma_{W}^{2}$$ 11

where δ­(*i*, *j*) is one when *i* = *j* and zero otherwise, while σ_W_^2^ is the variance hyperparameter of the kernel. Given the experimental nature of the data explored in this work and the corresponding expected degree of uncertainty, a White noise kernel was employed alongside the main kernels mentioned above.

Normalizing data is a common practice in the machine learning literature and is particularly useful in this work given the GP assumptions made above, namely that of a null mean function and kernels with single length scales. Normalizing the labels ensures a mean of zero while normalizing the features ensures that different types of variables (sigma profiles and temperature, for instance) possess the same scale and can thus be treated with the same length scale. In this work, two common data normalization techniques that have been found to work well for physicochemical property predictions with GPs from sigma profiles,^24^ namely standardization and log-standardization, were employed:

$$z^{\prime }=\frac{z-\left\langle z\right\rangle }{s_{z}}$$ 12

$$z^{\prime }=\frac{\log\left(z\right)-\left\langle \log\left(z\right)\right\rangle }{s_{\log\left(z\right)}}$$ 13

where *z* represents either features or labels and *z*′ is the transformed (i.e., normalized) variable, while ⟨ · ⟩ and *s* represent the mean and standard deviation of the untransformed variable. In cases where variables contain zeros (e.g., certain sigma profile values), a small buffer of 10^–3^ was added to *z* in eq 13.

All GP-related calculations were performed using the Python packages TensorFlow^34^ (V. 2.12.1) and GPFlow^33^ (V. 2.9.2). The three main kernels mentioned above were tested (eqs 8–10), always supplemented with the White noise kernel (eq 11). Thus, each GP model possessed between three to four hyperparameters (σ^2^, *l,* α*, σ*_W_^2^). These were optimized by maximizing the log marginal likelihood of each GP using the L-BFGS-B algorithm;^35^ as implemented in the Python package SciPy^36^ (V. 1.14.1).

### 2.3 DES Preparation

The chemical compounds experimentally used in this work to prepare DESs are listed in Table S1, along with their CAS numbers, purity, and suppliers. DESs at specific mole ratios were prepared by weighting the appropriate amount of each precursor into glass vials containing a stir bar and heating them in a paraffin bath at 80 °C with constant stirring for approximately 2 h, or until a clear and colorless liquid was formed. Mixtures that remained solid or turned solid upon equilibration at room temperature were discarded, as will be discussed. Densities and viscosities for liquid mixtures were measured using an SVM 3001 Anton Paar viscometer-densimeter. For the case of high-viscosity DESs, a cone–plate rotating springless viscometer, Lamy RM 100 CP-2000 PLUS, was employed.

### 2.4 Viscosity and Density Measurements

The viscosities and densities of the prepared DES samples were determined at atmospheric pressure and a temperature of 298.15 K. These measurements were carried out using an Anton Paar SVM 3001 viscometer-densimeter, with a temperature uncertainty of ±0.03 K, an absolute density uncertainty of 1 × 10^–4^ g·cm^–3^, and a relative viscosity uncertainty of 0.35%. For DESs exhibiting high viscosities (above 1000 cP), viscosity measurements were performed using a Brookfield DV-I cone–plate rotational viscometer. Data were collected over a 5 min period at 298.15 K, with shear rates corresponding to torque speeds ranging from 0 to 230 s^–1^. Regardless of the approach used, all measurements were performed in triplicate and are reported in this work as mean values accompanied by the corresponding standard deviations.

### 2.5 Tribological Tests

Tribological tests were performed using a TRB3 tribometer (Anton Paar, Graz, Switzerland) in a reciprocating ball-on-flat configuration at a room temperature of roughly 25 °C and a relative humidity of approximately 45%. The tribological pairs consisted of Si spheres (6 mm diameter) and Si plates. The sphere was mounted on the tribometer arm, while the Si substrate was secured to a metallic container using adhesive. Approximately three drops of liquid were applied to ensure full surface coverage.

The amplitude of the reciprocal movement of the counter body was 5 mm. A load of 1 N was used during the experiments (100 cycles, totaling a sliding distance of 1.00 m) to reduce surface wear (maximum contact pressure, *P*_max_, of 0.3 GPa). In these tests, the sliding velocity ranged from 1 to 20 mm/s. Both the spheres and plates were cleaned with ethanol and subjected to ultrasonic cleaning for 10 min before each test. A data acquisition rate of 50 Hz was used, and the results were analyzed with the TriboX software. The coefficient of friction (CoF) values were reported. As a reference, an initial test was conducted using the tribology reference oil (Part No. 228558, Anton Paar TriTec SA), with a viscosity of ∼55 cP at 40 °C and density of 0.860 g/cm^3^ at 20 °C. All measurements were performed in triplicate to ensure reliability.

## 3 Results and Discussion

The main results of this work are presented and discussed in this section, namely the data sets employed, the GP regression results, and the characterization and exploration of the DES design space toward obtaining novel lubricants.

### 3.1 Data Sets

All DES physicochemical properties (densities, viscosities, and melting temperatures) were taken from the publicly available database published by Odegova et al.^37^ This data set contains densities and viscosities as a function of DES composition and temperature, and melting temperature as a function of DES composition. Ambiguously named or duplicated compounds were removed, along with those that could not be treated with the DFT level of theory mentioned in Section 2.1. This resulted in an extensive database containing 398 unique DES combinations (different combinations of precursors, yielding binary or ternary systems) in the density data set, 412 in the viscosity data set, and 351 in the melting temperature data set. Considering unique mixtures (rather than unique DES combinations; different compositions count as different mixtures), the density and viscosity databases contain 748 and 754 unique points, respectively. The list of precursors includes inorganic compounds, salts, carboxylic acids, alcohols, aromatics, terpenes, amines, amides, esters, halogenated compounds, among many other families of compounds. Overall, across different DES combinations, compositions, and temperatures, there are 3985, 4197, and 2003 independent data points in the density, viscosity, and melting temperature data sets, respectively.

Following best practices in the machine learning community,^21^ each property data set was split into training (70%), validation (20%), and testing (10%) subsets. This procedure, known as a three-way hold-out, prevents overfitting and ensures that machine learning models can generalize to unseen features. Although GPs do not require hyperparameter tuning with a validation or testing set (unlike, for instance, neural networks), different kernels and normalization approaches were tested in this work. This was accomplished by fitting different models against the training set and evaluating their performance against the validation set. Normalization parameters (means and standard deviations) were derived exclusively from the training data sets to prevent data leakage between the training, validation, and testing sets. Once the optimal combination of kernel and normalization techniques was identified, the final models were tested against the testing set. To ensure that training, validation, and testing sets contain data representative of the full property data sets, stratified sampling was used. All data sets and their training-validation-testing splits are summarized in Table 1, with histograms reported in Figure 1. Note how the distributions of the properties across training, validation, and testing sets remain consistent with the corresponding distribution of the full property data set.

**Table 1:** Summary of the Training, Validation, and Testing Sets of the DES Property Datasets Explored in This Work, Including Their Sizes and Ranges

	training set		validation set		testing set	
data set	size	range	size	range	size	range
density/g·mL^–1^	2797	0.81–1.81	789	0.82–1.80	399	0.83–1.80
viscosity/cP	2946	4·10^–3^-9·10^5^	831	2·10^–2^-8·10^6^	420	4·10^–2^-4·10^5^
melting temp. /K	1405	96–574	397	78–575	201	142–573

**Figure 1:**
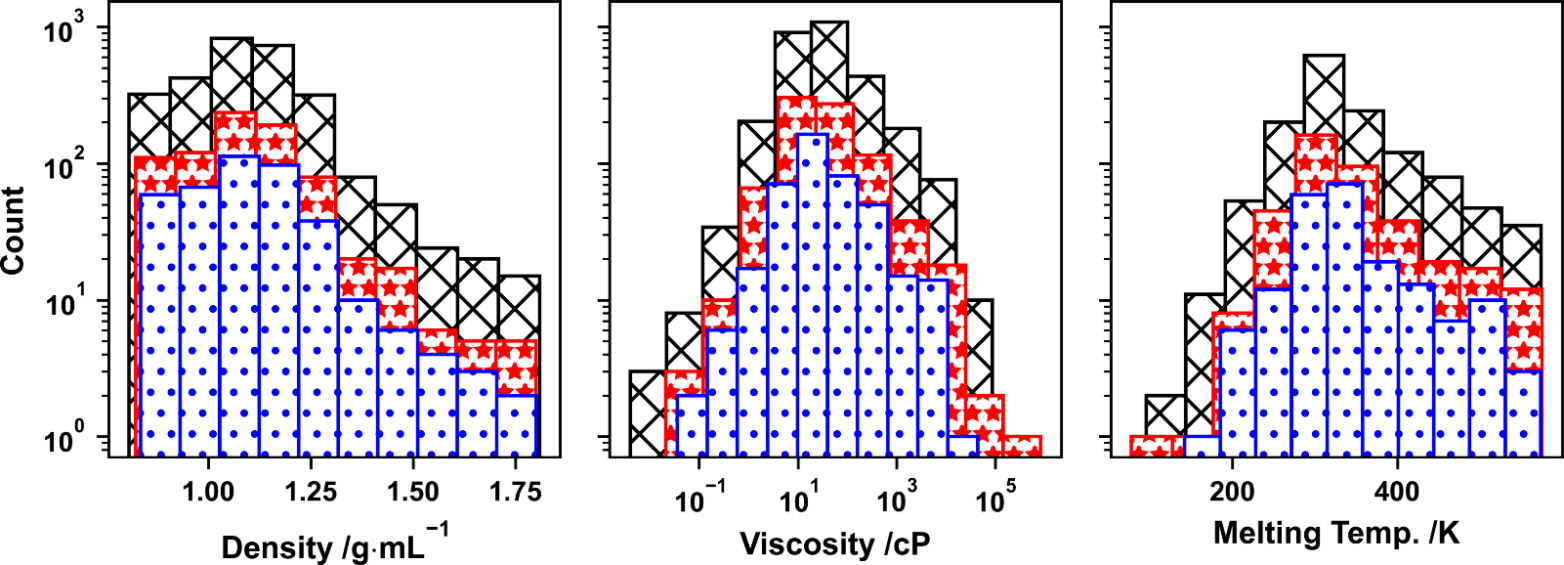
Count histograms for the training (black crosses), validation (red stars), and testing (blue circles) sets of the physicochemical properties studied in this work (density–left panel, viscosity–middle panel, melting temperature–right panel).

### 3.2 GP Regression

As explained above, benchmark tests were conducted to determine the optimal kernel (RBF, RQ, or M32) and data normalization approaches (none, standardization, or log-standardization) to be used in this work. To do so, a new GP was trained on the training set for each property, considering each possible combination of kernel and normalization method (totaling 9 combinations for each property), and subsequently evaluated on the validation set. These results (coefficient of determination for the validation set) are reported in Table S2 of Supporting Information. Interestingly, changing kernels and normalization techniques did not significantly affect GP performance in the prediction of density and melting temperature. In contrast, these choices had a substantial impact on GP performance for viscosity prediction, where only label log-standardization yielded viable models. This is related to the distribution of viscosity data, as illustrated in Figure 1, which spans several orders of magnitude.

No single combination of kernels and normalization techniques yielded the best results (as quantified by validation coefficients of determination) across all three properties studied. This is not surprising and has been observed in the past.^24^ Regarding kernel choice, average validation coefficients of determination (across all normalization techniques and properties) of 0.94, 0.94, and 0.95 were obtained for the RBK, RQ, and M32 kernels, respectively. Similarly, average values of 0.98, 0.99, and 0.99 were obtained for no feature normalization, feature standardization, and feature log-standardization. Finally, only label log-standardization produced usable models for viscosity. As such, the M32 kernel was selected to be used in this work, along with log-standardization for both features and labels. This kernel/normalization set was employed to perform the final fitting of the GP models for each property data set. The results of this fitting, including performance on the training, validation, and testing sets, are depicted in Figure 2.

**Figure 2:**
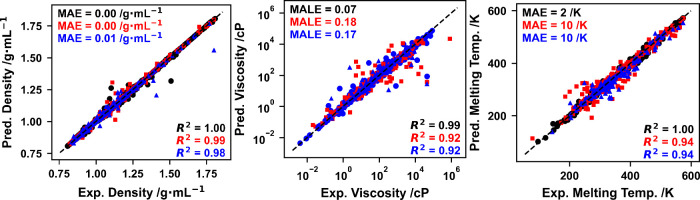
GP-predicted (*y*-axis) vs experimental (*x*-axis) density (left panel), viscosity (middle panel), and melting temperature (right panel) for the training (black circles), validation (red squares), and testing (blue triangles) sets of the physicochemical properties studied in this work (density–left panel, viscosity–middle panel, melting temperature–right panel). Mean absolute errors (MAEs), mean absolute logarithmic errors (MALEs; for viscosity only), and coefficients of determination for each set are also included.

As depicted in Figure 2, the density GP model demonstrates exceptional predictive performance across all data sets, with coefficients of determination of 1.00, 0.99, and 0.98 for the training, validation, and testing sets, respectively, indicating an almost perfect agreement between experimental and predicted values. The viscosity GP model, analyzed on a logarithmic scale due to the wide range of values (roughly 9 orders of magnitude), shows satisfactory performance with coefficients of determination of 0.98, 0.92, and 0.92. Finally, the melting temperature GP model exhibits strong performance, with coefficients of determination of 1.00, 0.94, and 0.94, and consistent low MAE values across data sets. Overall, all three models effectively capture the relationship between DES sigma profiles, composition, and temperature with experimental physicochemical properties, demonstrating high generalization and reliability, and yielding predictive tools suitable for the design of novel DESs. This is a remarkable result, particularly considering that both binary and ternary DESs are being used.

It is worth noting that various machine learning models capable of predicting the properties examined in this work for DESs have already been reported in the literature. For density, Abdollahzadeh et al.^38^ analyzed 149 DESs, achieving testing set coefficients of determination of up to 0.99 utilizing different ML models. Wang et al.^39^ attained similar performances with random forests. Regarding viscosity, Mohan et al.^20^ applied gradient boosting (CatBoost) to 672 binary DES samples, comprising 4949 data points over a temperature range of 278.15–385.25 K, achieving an excellent coefficient of determination on the testing set of 0.99. Roosta et al.^40^ investigated viscosity across 305 DES combinations and 2533 data points within a slightly narrower temperature range (277.15–373.15 K), achieving similar performances. Additional studies by Shi et al.^41^ and Liu et al.^42^ also investigated viscosity predictions, albeit with fewer samples or incomplete variable range information, reaching R^2^ higher than 0.98. Finally, Ayres et al.^22^ employed extreme gradient boosting to predict DES melting temperatures using 237 DES combinations and 1648 data points, achieving an *R* ^2^ of 0.976.

While better-performing ML models have been reported in the literature (albeit in many cases with less strict data splitting, training, and evaluation procedures), as listed above, the GPs developed in this work offer several distinct advantages. First and most importantly, the models developed for density and viscosity can handle ternary DESs, while most models listed above can only predict properties for binary mixtures. In fact, the data sets used in this work for density and viscosity include 287 and 891 independent ternary DES data points, respectively. This capability allows, for example, the prediction of the effects of low molecular weight additives on the properties of DESs (of particular significance for viscosity, as explained in the Section 1) Furthermore, due to the construction procedure employed for DES sigma profiles (see Section 2.1), the GP models can accommodate DESs with any composition rather than being limited to specific mole ratios. Moreover, the data sets used here encompass a much greater chemical diversity, ranging from type I DESs (based on metal salts) to type V DESs (based solely on nonionic compounds). Even ionic liquids are included. Finally, the training and usage of GPs are significantly less computationally expensive compared to several other machine learning methods, such as neural networks.

Notwithstanding the previous paragraphs, the most relevant performance comparison can be obtained by examining the results reported by Odegova et al.,^37^ given that the data sets used are identical. Odegova et al.^37^ reported overall coefficients of determination of 0.91, 0.60, and 0.78 for the density, viscosity, and melting temperature data sets, respectively. As such, the GP performances reported in this work represent a tremendous improvement, attributable not only to the inherent predictive capability of GPs—a class of ML models often overlooked in the field of materials design—but also to the utilization of sigma profiles as molecular features. Due to the extensive chemical information encoded in sigma profiles, including polarity and potential for intermolecular interactions, which are key parameters governing the formation and properties of DESs, sigma profiles bridge the knowledge gap between features and labels more easily and with fewer data points or more scarce data sets.^24,43^ This enables machine learning models to achieve superior predictive performance compared to other types of molecular features.

### 3.3 DES Discovery

Sigma profiles represent a mathematically continuous space. Thus, and as reported before for organic compounds,^24^ sigma profiles can be conceptualized as a digital space, navigable using pretrained GP models. Given that DES sigma profiles, as defined in this work, consist of vectors of size 183, the dimensionality of this digital space is 183. Unfortunately, visualizing a mathematical space with more than three dimensions (let alone 183) is challenging. However, high-dimensional spaces can be approximated with two dimensions using Principal Component Analysis (PCA). This method constructs new variables, known as principal components, which are approximate linear combinations of the original features and exhibit no correlation with one another. In essence, PCA enables the visualization of complex, high-dimensional data sets by projecting them into an approximate lower-dimensional space.^44^ To visualize the DES space covered in the previous section, the GP predictions reported in the previous section are now depicted in Figure 3 as three-dimensional surface plots (*z*-axis) against the sigma profile PCA space (*xy*-plane). PCA was performed based on all DES combinations included in the databases studied, utilizing the method published by Tipping and Bishop.^45^

**Figure 3:**
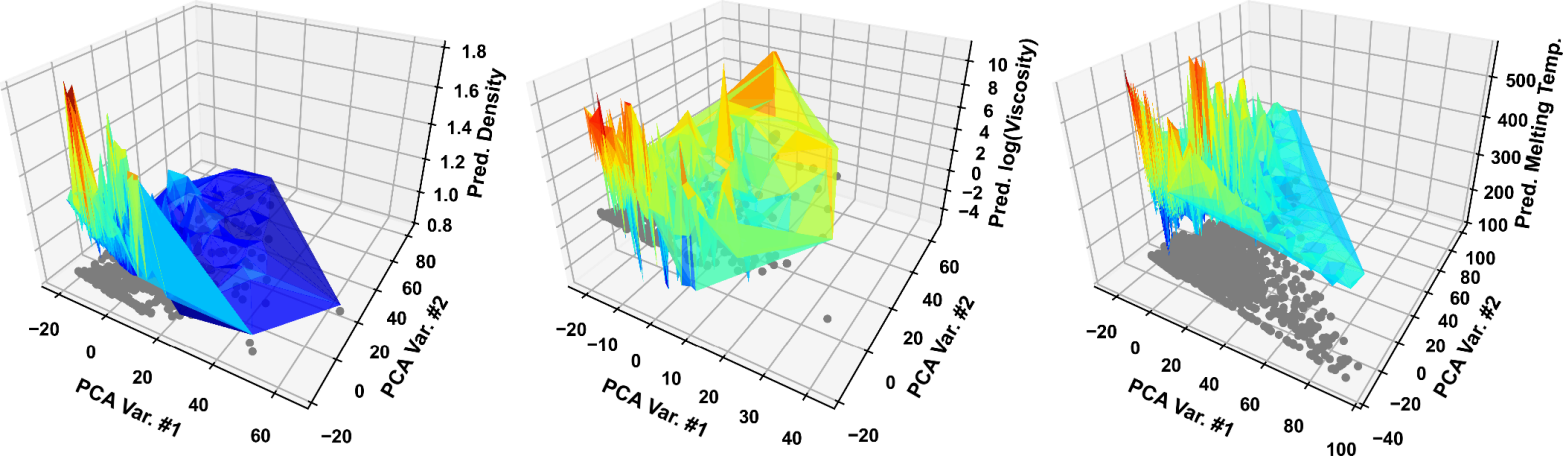
GP-predicted density (left panel), viscosity (middle panel), and melting temperature (right panel) for the DESs present in the database studied in this work, represented as three-dimensional surfaces superimposed on the two-dimensional abstract PCA space. Each gray circle represents the 2-D PCA point of a unique 183-D DES sigma profile. For density and viscosity, all temperature-dependent results are superimposed in the same *xy*-plane point.

The depictions in Figure 3 are particularly useful to highlight uncharted areas within the DES space. Despite the extensive database used in this work, a significant portion of the DES space remains unexplored, as indicated by the absence of data points across most of the *xy*-planes in Figure 3. This should come as a surprise given that the database used here contains 342 individual DES precursors. Even when considering only equimolar DES mixtures, this should yield over a million unique ternary DES combinations. In stark contrast, the density and viscosity data sets contain only approximately 750 unique DES mixtures, respectively (and, thus, roughly 750 unique sigma profile points). The most comprehensive data set, that of the melting temperature, still contains only around 2000 independent sigma profile points, which is considerably less than one million. Overall, Figure 3 underscores the significant gap in DES knowledge and data in the literature, as most studies typically focus on well-known DES combinations (e.g., choline chloride/urea or choline chloride/glycerol) at specific mole ratios, rather than properly exploring the full DES design space.

In general, the viscosity and density of a lubricant play a crucial role in its performance and are strongly influenced by the specific application and operating conditions.^46^ Viscosity, in particular, affects the ability of lubricants to maintain film thickness, directly impacting lubrication efficiency and durability. High-density/high-viscosity lubricants tend to offer superior film strength and protection under heavy loads, making them suitable for industrial machinery and equipment operating under demanding conditions. Conversely, low-density/low-viscosity lubricants minimize internal resistance and friction, enhancing energy efficiency, an increasingly important criterion in modern lubrication strategies.^47^ The trend toward low-viscosity lubricants aims to reduce frictional losses, as excessively viscous fluids can lead to power losses and increased operational costs. For DESs, viscosity is primarily governed by the nature of their components, molar ratio, temperature, and water content. Consequently, the search for low-viscosity DESs that retain high lubrication performance has become a key objective in lubricant design.

In this work, both types of DES lubricants (high viscosity/high density and low viscosity/low density) were considered. There is an infinite number of possible DESs that can be envisioned from various precursor combinations and relative compositions. Gradient search techniques could be used to navigate this space using fine composition grids,^24^ but precursors are limited to those readily available in the laboratory or easily procured, sourced, or synthesized. Thus, a more pragmatic approach was adopted in this work by considering only binary 1:1 combinations of the 342 precursors, at a temperature of 298 K, contained in the database, yielding a manageable set of 58,311 unique combinations.

Considering the vast amount of DES predictions depicted in Figure 4, potential DES lubricants were selected considering the availability of precursors in our laboratory. Two sets were chosen: one set aimed at minimizing both density and viscosity with DESs predicted to be liquid below 275 K, and another set aimed at maximizing both density and viscosity, provided their predicted melting temperature was below 300 K. The selected GP-discovered DESs, along with their predicted and experimental properties (experimentally measured in this work), are reported in Table 2. Curiously, for the room-temperature-liquid, high density, high viscosity DESs, GPs tended to rely heavily on precursors based on ionic liquids (or salts with ions traditionally associated with the field of ionic liquids).

**Figure 4:**
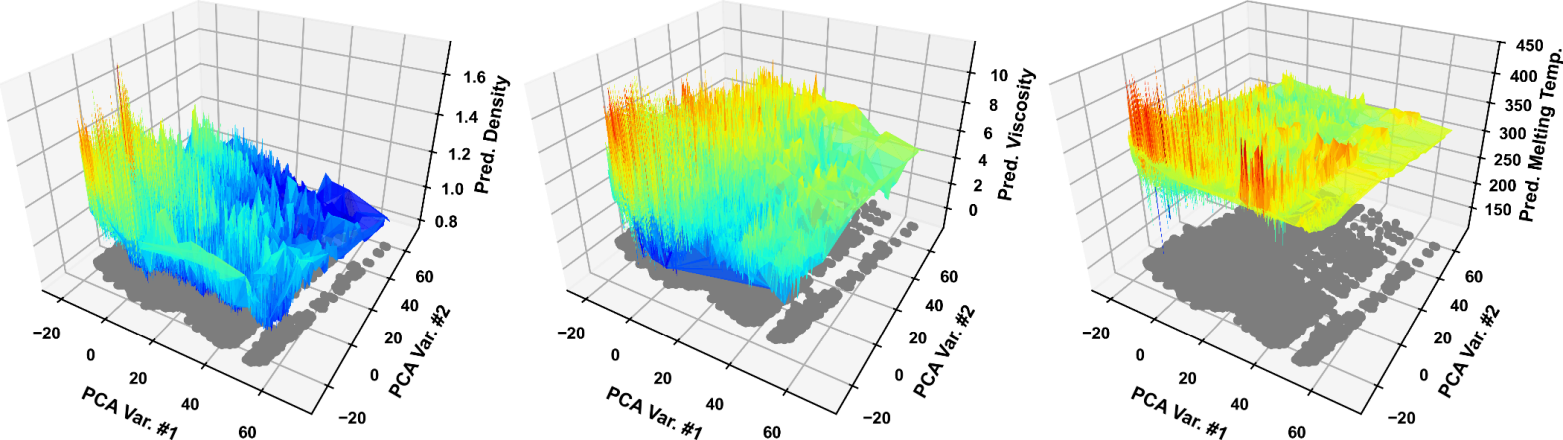
GP-predicted density at 298 K (left panel), viscosity at 298 K (middle panel), and melting temperature (right panel) for all unique binary DES combinations (at 1:1 mol ratios) out of the precursors in the database studied in this work, represented as three-dimensional surfaces superimposed on the two-dimensional abstract PCA space. Each gray circle represents the 2-D PCA point of a unique 183-D DES sigma profile.

**Table 2:** List of DESs Experimentally Prepared in This Work (in a 1:1 mol Ratio), along with Their GP Predicted and Experimental Densities and Viscosities, Measured in This Work as Detailed in Section 2 ^a^

		predicted (298 K)		experimental (298 K)	
HBA	HBD	ρ /g·mL^–1^	η /cP	ρ /g·mL^–1^	η /cP
1-butanol	propionic acid	0.89	4.0	0.887 ± 0.009	1.849 ± 0.001
eucalyptol	octanoic acid	0.90	5.5	0.915 ± 0.008	4.602 ± 0.002
propionic acid	heptanoic acid	0.98	4.3	0.93 ± 0.01	1.948 ± 0.001
camphor	octanoic acid	0.93	10.0	0.93 ± 0.01	8.402 ± 0.006
m-cresol	3-amino-1-propanol	1.00	102	1.03 ± 0.01	102.8 ± 0.7
[N_1,1,1,Bz_]Cl	lactic acid	1.09	652	1.12 ± 0.02	35.94 ± 0.03
[C_4_C_1_im]Br	glucose	1.37	6294	n.a.	^b^(6 ± 1)·10^4^
[N_3,3,3,3_]Br	lactic acid	1.09	262	n.a.	^b^(2.7 ± 0.5)·10^3^

a Abbreviations are defined in Table S1.

b Large uncertainties.

The differences between predicted and experimental values are minimal in the case of density, but there are considerable differences between the predicted and experimental viscosities of the system [N_1,1,1,Bz_]­Cl-lactic acid. There are also severe differences for the systems [C4C1im]­Br-glucose and [N_3,3,3,3_]­Br-lactic acid, but those exhibit large uncertainties (see Figure S2). Considering the current trend toward low-viscosity lubricants, which aim to minimize frictional losses, since highly viscous fluids may result in increased power consumption and higher operational costs, the four DES with the lowest viscosities were selected for measurements of coefficients of friction (CoF). Additionally, a reference oil (see Section 2) was also included for comparison.

The CoF results, presented in Figure 5 and Table S3 of the Supporting Information, show significant variations in the tribological performance of the systems tested, emphasizing the important role of DES composition in lubrication efficiency. As expected, the absence of lubricant resulted in the highest CoF (∼0.17), reinforcing the fundamental role of lubrication in reducing frictional losses. The reference oil effectively reduced friction, serving as a baseline for evaluating the performance of the DES-based systems.

**Figure 5:**
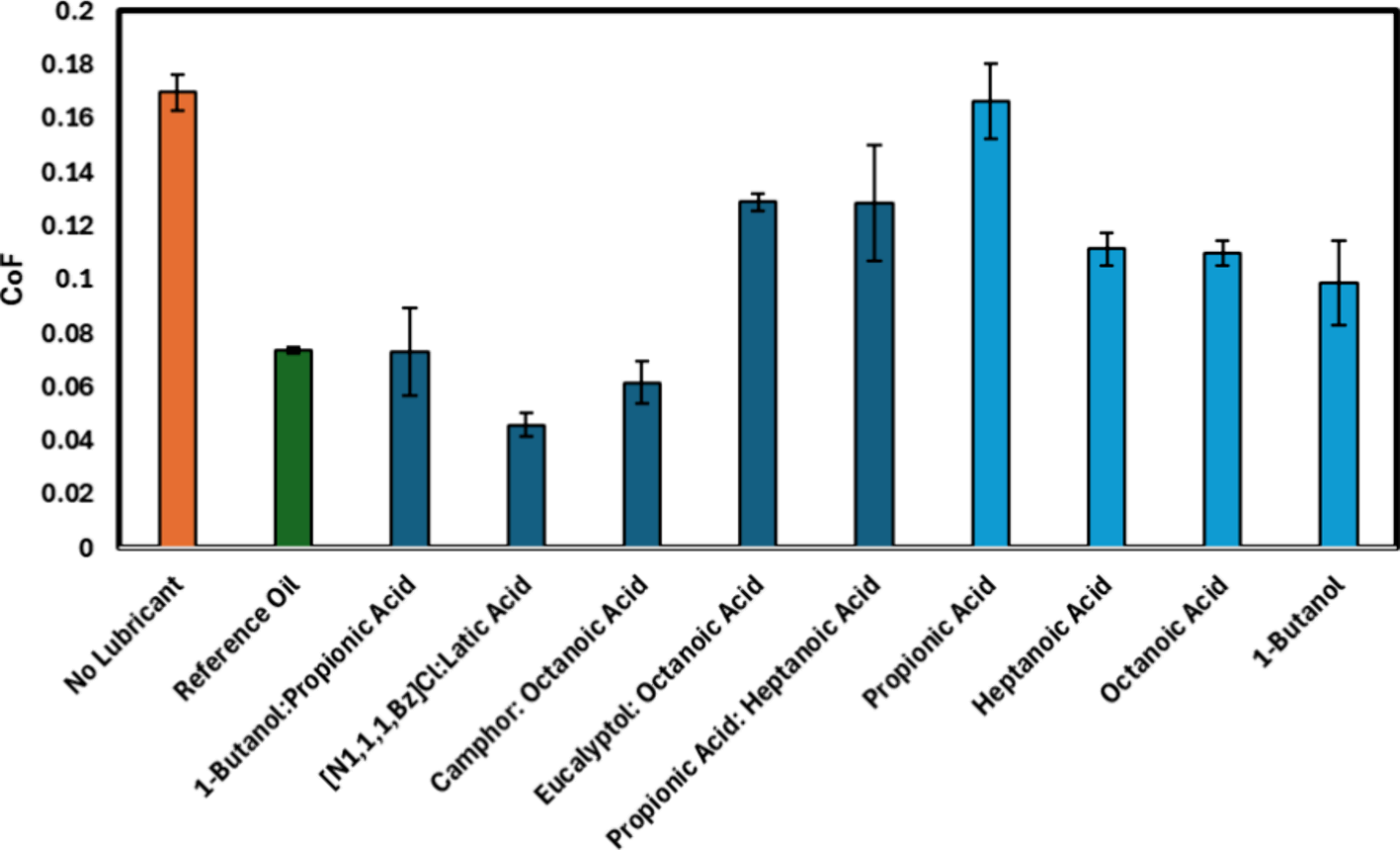
Coefficient of friction (CoF) values obtained for: (orange square) no lubricant, (green square) reference oil, (dark blue square) various GP-discovered DESs in a 1:1 molar ratio, and (bright blue square) their individual precursors (when liquid), measured at room temperature (∼25 °C) and relative humidity (∼45%).

Among the DESs tested, the Camphor:Octanoic Acid system exhibited a notably low CoF, achieving a 16.5% reduction compared to the reference oil. This performance highlights the potential of terpene-based DES for tribological applications. The rigid and nonpolar structure of camphor likely promotes the formation of an ordered molecular arrangement at the sliding interface, while octanoic acid acts as an effective surface-active agent, facilitating the development of a stable boundary film under load. Another notable formulation is [N_1_,_1_,_1_,_Bz_]­Cl:Lactic Acid, which presented the lowest CoF among all tested systems, a reduction of 37.9% relative to the reference oil. Interestingly, although this DES presents a relatively high density (1.09 g·cm^–3^) and its viscosity was predicted to be high (652 cP), it exhibited a substantially lower experimental viscosity (36 cP), which is consistent with its favorable lubrication performance.

An interesting aspect comes from the comparison between the DES and their individual components (HBA and HBD). For example, the 1-Butanol:Propionic Acid mixture showed a CoF significantly lower than either of its pure components. While pure 1-butanol and propionic acid exhibited CoFs around 0.11–0.13, the DES achieved a value close to 0.07, clearly indicating a synergistic effect. This nonadditive behavior suggests that the eutectic structure of the mixture alters molecular interactions in a favorable way, leading to more effective tribofilm formation than would be expected from the individual components.^48^

These findings demonstrate that the tribological performance of DES results from a complex interaction between molecular structure, polarity, viscosity, and self-organization capacity. They highlight the potential for the rational design of tribologically active DES, where the strategic selection of HBA and HBD components can lead to emergent properties not predictable from the pure substances, underscoring the importance of the GP approach used in this study.

## 4 Conclusions

This study successfully demonstrated the potential of DESs as viable alternatives to conventional mineral-based lubricants. This study also developed and reported a robust framework to predict the physicochemical properties of DESs using GPs. The integration of sigma profiles and advanced normalization techniques facilitated accurate predictions across a diverse range of compositions and temperatures, achieving high reliability for the physicochemical properties studied (density, viscosity, and melting temperature). Through comparison with existing literature, GPs demonstrated superior performance, particularly in terms of generalizability and coverage of extensive data ranges. This allowed for the estimation of DES properties over a wide range of possible precursor combinations.

Building on the robust GP framework developed in this study, which accurately predicted the physicochemical properties of DESs, we selected a subset of DESs with exceptionally low predicted viscosities for tribological evaluation. The results from these tribological tests confirmed the potential of DESs as viable alternatives to conventional lubricants, with a remarkable reduction in the coefficient of friction of up to 37.9% compared to the reference oil. These results highlight the potential of DES for real-world applications, offering a promising avenue for the development of advanced lubricants.

An advantage of GPs, not mentioned thus far, is their stochastic nature. This means that GPs can function as laboratory companions, and their uncertainty can be used to guide the acquisition of new data to improve the model, a framework already employed to measure the viscosity of ternary DESs.^18^ Such workflows can greatly benefit from pretrained GP models as starting points, particularly the model published here. Finally, while the computation of sigma profiles may represent a bottleneck in the workflow developed in this work, due to both the commercial nature of the quantum chemistry software packages used in this work and the computational expense of performing these calculations, it is worth noting that open-source software is available to compute sigma profiles,^49^ as well as ML models capable of predicting sigma profiles from molecular structures.^50^

## Supplementary Material



## Notes

Sigma profiles used in this work, information about the chemical compounds used experimentally, and additional computational and experimental results (PDF)
