# Supplementary material for: Artificial Intelligence in the Discovery of Deep Eutectic Solvents with Lubricant Applications
Source: ACS Omega. 2025 Sep 10;10(37):43024–33. doi: 10.1021/acsomega.5c05944 (PMC12461321; doi:10.1021/acsomega.5c05944)
Supplement: Supplementary file 1 [file ao5c05944_si_001.pdf]

Supporting Information

Artificial Intelligence in the Discovery of Deep Eutectic Solvents with Lubricant Applications

João P. Santos,[1] Filipe H. B. Sosa,[1*] Dinis O. Abranches,[1*] João A. P. Coutinho[1]

[1]CICECO – Aveiro Institute of Materials, Department of Chemistry, University of Aveiro, 3810-193, Portugal

Corresponding authors: jdinis@ua.pt | filipesosa@ua.pt

Contents

S1. Figures 2
Figure S1. 2
Figure S2. 2
S2. Tables 3
Table S1. 3
Table S2. 4
Table S3. 5

S1. Figures

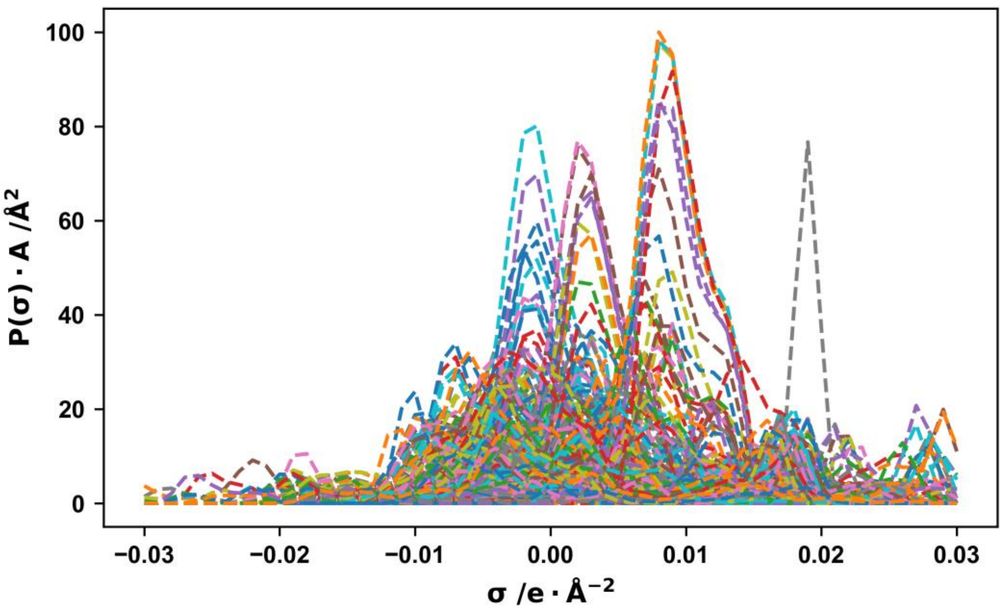

Figure S1. *Sigma profiles for all DES components investigated in this work. Note that a σ range from -0.03 e·Å to 0.03 e·Å in intervals of 0.001 e·Å was employed.*

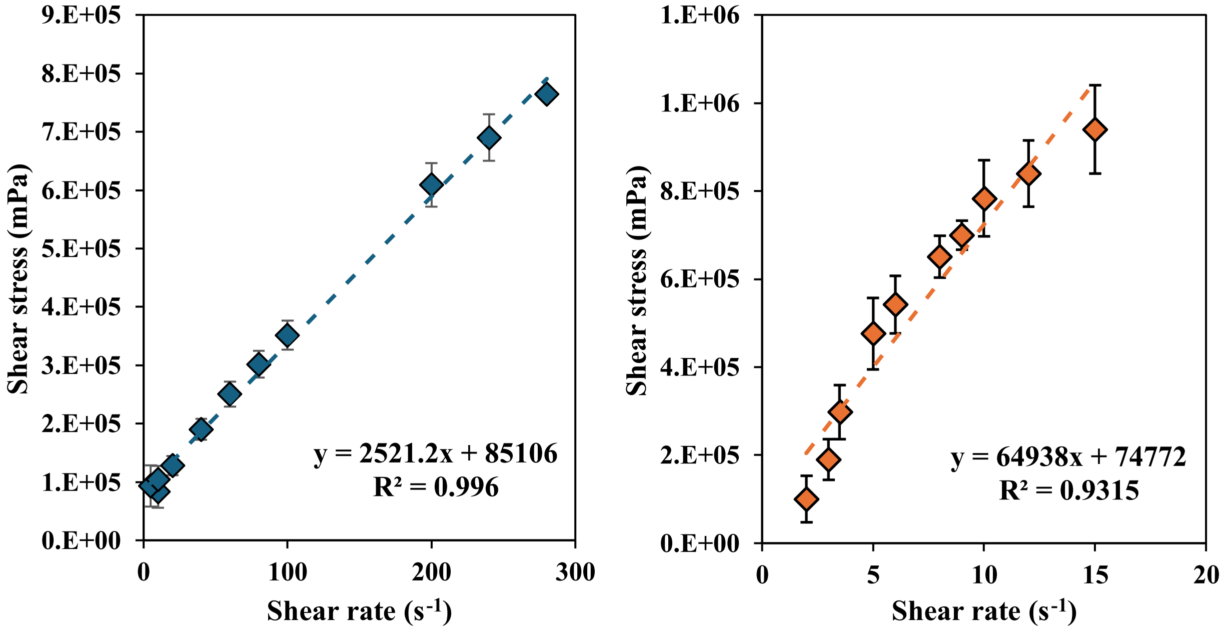

Figure S2. *Shear stress as a function of shear rate, at 25 ºC, for [N3,3,3,3]Br:Lactic Acid 1:1 mole ratio (left panel) or [C_4C_1im]Br:Glucose 1:1 mole ratio (right panel).*

S2. Tables

Table S1. *Name, CAS number, supplier, and purity of the chemical compounds experimentally used in this work.*

Name	CAS Number	Supplier	Purity
1-Butanol	71-36-3	VWR Chemicals	99.5%
3-Amino-1-propanol	156-87-6	TCI	99%
Propionic Acid	79-09-4	Merck	99%
Heptanoic Acid	111-14-8	Aldrich	99%
Octanoic Acid	124-07-2	TCI	98%
Lactic Acid	79-33-4	Sigma-Aldrich	92%
Eucalyptol	470-82-6	Alfa Aesar	99%
Camphor	76-22-2	Alfa Aesar	98%
m-Cresol	108-39-4	Acros Organics	99%
Glucose	50-99-7	Thermo Scientific	99%
Benzyltrimethylammonium Chloride - $[N_{1,1,1,Bz}]Cl$	56-93-9	Acros Organics	98%
1-Butyl-3-methylimidazolium bromide - $[C_4C_1im]Br$	85100-77-2	Iolitec	99%
Tetrapropylammonium bromide - $[N_{3,3,3,3}]Br$	1941-30-6	Aldrich	98%
Reference Oil (Part No. 228558)	-	Anton Paar TriTec SA	-

Table S2. *GP performance (validation set coefficient of determination) in the prediction of density, viscosity, and melting temperature. GP fitting relied on a white noise kernel combined with either an RBF, RQ, or M32 kernel. Three types of normalization approaches for both features (F. Norm.) and labels (L. Norm.) were tested, namely no normalization, standardization (Stand.), and log-standardization (Log-Stand.).*

Kernel	F. Norm.	L. Norm.	Density	Viscosity	Melting T.
RBF	None	None	0.98	*	0.91
		Stand.	0.99	*	0.90
		Log-Stand.	0.98	0.89	0.90
	Stand.	None	0.96	*	0.92
		Stand.	0.99	*	0.93
		Log-Stand.	0.99	0.92	0.92
	Log-Stand.	None	0.99	*	0.88
		Stand.	0.99	*	0.92
		Log-Stand.	0.99	0.90	0.91
RQ	None	None	0.98	*	*
		Stand.	0.98	*	0.89
		Log-Stand.	0.98	0.61	0.91
	Stand.	None	0.99	*	0.94
		Stand.	0.99	*	0.93
		Log-Stand.	1.00	0.86	0.93
	Log-Stand.	None	0.99	*	0.94
		Stand.	0.99	*	0.94
		Log-Stand.	0.99	0.92	0.94
M32	None	None	0.99	*	0.92
		Stand.	0.99	*	0.91
		Log-Stand.	0.99	0.89	0.91
	Stand.	None	0.99	*	0.94
		Stand.	0.99	*	0.94
		Log-Stand.	0.99	0.90	0.93
	Log-Stand.	None	0.99	*	0.95
		Stand.	0.99	*	0.94
		Log-Stand.	0.99	0.92	0.94

*Negative values or unable to fit the GP model.

Table S3. Coefficient of friction (CoF) values and corresponding standard deviations obtained for: no lubricant, reference oil, various DESs (1:1 molar ratio) identified via GP, and their individual precursors (when in liquid state), measured at room temperature (~25 °C) and relative humidity (~45%).

Solvent	Coefficient of friction (CoF)
No Lubricant	0.170±0.007
Reference Oil	0.074±0.001
1-Butanol:Propionic Acid	0.073±0.016
BAC:Latic Acid	0.046±0.004
Camphor: Octanoic Acid	0.062±0.008
Eucalyptol: Octanoic Acid	0.129±0.003
Propionic Acid: Heptanoic Acid	0.129±0.022
Propionic Acid	0.166±0.012
Heptanoic Acid	0.111±0.006
Octanoic Acid	0.110±0.005
1-Butanol	0.099±0.016
